# Chronic post-COVID neuropsychiatric symptoms persisting beyond one year from infection: a case-control study and network analysis

**DOI:** 10.1038/s41398-024-02978-w

**Published:** 2024-06-19

**Authors:** Steven Wai Ho Chau, Timothy Mitchell Chue, Rachel Ngan Yin Chan, Yee Lok Lai, Paul W. C. Wong, Shirley Xin Li, Yaping Liu, Joey Wing Yan Chan, Paul Kay-sheung Chan, Christopher K. C. Lai, Thomas W. H. Leung, Yun Kwok Wing

**Affiliations:** 1 Department of Psychiatry, Faculty of Medicine, The Chinese University of Hong Kong, Hong Kong, China; 2 Li Chiu Kong Family Sleep Assessment Unit, Department of Psychiatry, Faculty of Medicine, The Chinese University of Hong Kong, Hong Kong, China; 3 Department of Social Work and Social Administration, Faculty of Social Science, The University of Hong Kong, Hong Kong, China; 4 Department of Psychology, Faculty of Social Science, The University of Hong Kong, Hong Kong, China; 5 Center for Sleep and Circadian Medicine, The Affiliated Brain Hospital of Guangzhou Medical University, Guangzhou, Guangdong China; 6 Department of Microbiology, Faculty of Medicine, The Chinese University of Hong Kong, Hong Kong, China; 7 Division of Neurology, Department of Medicine and Therapeutics, Faculty of Medicine, The Chinese University of Hong Kong, Hong Kong, China

**Keywords:** Diseases, Psychiatric disorders

## Abstract

Our study aims to delineate the phenotypes of chronic neuropsychiatric symptoms among adult subjects recovering from their first COVID that occurred more than one year ago. We also aim to explore the clinical and socioeconomic risk factors of having a high loading of chronic neuropsychiatric symptoms. We recruited a post-COVID group who suffered from their first pre-Omicron COVID more than a year ago, and a control group who had never had COVID. The subjects completed app-based questionnaires on demographic, socioeconomic and health status, a COVID symptoms checklist, mental and sleep health measures, and neurocognitive tests. The post-COVID group has a statistically significantly higher level of fatigue compared to the control group (*p* < 0.001). Among the post-COVID group, the lack of any COVID vaccination before the first COVID and a higher level of material deprivation before the COVID pandemic predicts a higher load of chronic post-COVID neuropsychiatric symptoms. Partial correlation network analysis suggests that the chronic post-COVID neuropsychiatric symptoms can be clustered into two major (cognitive complaints -fatigue and anxiety-depression) and one minor (headache-dizziness) cluster. A higher level of material deprivation predicts a higher number of symptoms in both major clusters, but the lack of any COVID vaccination before the first COVID only predicts a higher number of symptoms in the cognitive complaints-fatigue cluster. Our result suggests heterogeneity among chronic post-COVID neuropsychiatric symptoms, which are associated with the complex interplay of biological and socioeconomic factors.

## Background

Post-acute COVID syndrome (PACS), also known as ‘Long-COVID’, is a major global health concern [1]. While SARS-CoV-2 is primarily a respiratory virus, COVID is now recognised as a multisystem disease [2, 3]. Neuropsychiatric symptoms are among the most common non-respiratory symptoms both in the acute phase of COVID and PACS, such as mood and anxiety symptoms, sleep disturbance, fatigue and cognitive complaints [4]. Available evidence suggests that the risk of increased incidence in several neuropsychiatric conditions occurs not only within the first few months of SARS-CoV-2 infection, but can also extend up to a year beyond infection [5]. Recent studies have also shown changes in the brain post-SARS-CoV-2 infection. A longitudinal MRI study, for example, has demonstrated structural brain changes after SARS-CoV-2 infection [6]. MRI imaging studies have suggested grey matter or cortical changes among those suffering from PACS [7, 8]. The way SARS-CoV-2 infection affects brain health in the long term, however, is not well understood. Currently, the prevailing hypothesis links neuropsychiatric symptoms of PACS with neuroinflammation and prolonged immune dysregulation triggered by SARS-CoV-2 infection, and/or microvascular pathology secondary to endothelial dysfunction [9].

Significant knowledge gaps also exist regarding chronic neuropsychiatric sequelae after SARS-CoV-2 infection. First, most PACS studies have focused on the first year after SARS-CoV-2 infection, leaving questions about post-COVID symptoms that run a chronic course, which is arguably a major public health concern. Second, while many PACS studies have simply considered neuropsychiatric complaints as part of a unitary PACS syndrome, this approach may overlook the heterogeneity of diverse experiences under the PACS umbrella, as well as unique factors associated with different symptom groups. For example, while preliminary evidence suggests that clinical factors, such as infection severity and COVID vaccination, are predictive factors for PACS [10], socioeconomic factors and pre-existing mental illness are often neglected in the PACS literature. Third, there have been very few studies thus far that attempt to delineate the phenotypes of chronic neuropsychiatric aspects of PACS by exploring the relationship among subjective neuropsychiatric symptoms and corresponding quantitative mental and cognitive measures.

In response to these gaps in knowledge, our current study aims to use a symptom-based approach, supplemented by validated symptoms, health-related quality of life (HRQoL) measures and app-based cognitive tests, to delineate the phenotypes of chronic neuropsychiatric symptoms among young to middle-aged adult subjects recovering from their first, pre-Omicron strain SARS-CoV-2 infection that occurred more than one year ago. We also aim to explore the relationship and clustering among common chronic neuropsychiatric symptoms, and to test if these clusters are associated with different risk factors. We set out the following hypotheses: (1) Post-COVID subjects would have a higher level of neuropsychiatric symptoms, worse neurocognitive performance and worse HRQoL 1 year after being first infected by SARS-CoV-2 compared to control subjects who had never been infected by SARS-CoV-2; (2) Clinical factors such as the severity of the index infection, vaccination status, and socioeconomic status will be associated with the level of chronic neuropsychiatric complaints persisting beyond one year of the index infection among post-COVID subjects; (3) Chronic neuropsychiatric symptoms among post-COVID subjects will form clusters discoverable by network analysis.

## Methods

Subjects for the post-COVID group and the control group were concurrently recruited in the community from 24 August 2022 to 1 March 2023 via posters in hospitals, online advertisements via social media, and university email newsletters. All advertising material was available in both Chinese (in traditional characters) and English language. The inclusion criteria for the post-COVID group were as follows: (i) report history of a laboratory-confirmed SARS-CoV-2 infection which occurred before January 2022 (i.e. before the Omicron variant became dominant in Hong Kong) [11]; (ii) first SARS-CoV-2 infection occurred at least one year prior to the study, and; (iii) age between 18–65 years. Inclusion criteria for the non-COVID control group were as follows: (i) no history of SARS-CoV-2 infection as confirmed by lateral-flow test or PCR; and (ii) matched with the post-COVID group in terms of the following 5 characteristics: age, gender, ethnicity, pre-OVID medical and psychiatric comorbidities, and socioeconomic status (primarily measured by the locally developed Deprivation Index, which measures material deprivation and has been widely used for local public health research [12, 13]). All participants gave written informed consent either in person or via videoconference. We then instructed all participants to install a mobile phone app designed for this study. Using the app, participants completed (i) a set of questionnaires on their demographic information and socioeconomic and health status at two time points: December 2019, which was immediately before the COVID pandemic reached Hong Kong [11], and at the time of the assessment; (ii) a PACS symptoms checklist that comprised of 15 neuropsychiatric items and 26 non-neuropsychiatric items (see Supplementary materials for full checklist): the choice of items was based on results from earlier PACS studies [4], and we subsequently classified the severity of subjects’ acute COVID into three categories: (a) asymptomatic or mild, for subjects who did not have symptoms of pneumonia; (b) moderate, for subjects who had symptoms suggestive of pneumonia (i.e. presence of fever, cough, and shortness of breath) but did not require oxygen therapy; and (c) severe or critical, for subjects who had symptoms suggestive of pneumonia and required oxygen therapy and/or intensive care; (iii) a set of questionnaires on standardised mental health, sleep and HRQoL measures as follows: Patient Health Questionnaire-9 (PHQ-9), General Anxiety Disorder-7 (GAD-7), Impact of Event Scale-revised (IES-r) [14], Insomnia Severity Index (ISI) [15], Chalder Fatigue Scale (CFS) [16], CAGE screening questionnaire for alcoholism, Global Physical Activity Questionnaire (GPAQ) [17] and WHOQOL-BREF (excluding the environmental domain) [18]; (iv) a seven-day sleep diary; and (v) app-based cognitive tasks focusing on key domains (concentration, psychomotor speed and working memory) related to the phenomenon of ‘brain fog’ [19], which included the psychomotor vigilance test (PVT), digital symbols substitution test (DSST), N-back test, and alternate finger-tapping test (see Supplementary Method for test instructions and scoring methods). The application was available in Chinese(in traditional characters) or English depending on the subjects’ preference. Subjects who completed all assessments were compensated with HKD$150 (~USD$20) in supermarket coupons. The authors assert that all procedures contributing to this work comply with the ethical standards of the relevant national and institutional committees on human experimentation and with the Helsinki Declaration of 1975, as revised in 2008. All procedures involving human subjects were approved the Joint Chinese University of Hong Kong-New Territories East Cluster Clinical Research Ethics Committee (Ref.: 2022.362). The study protocol was registered on the ISTCTN registry on 8 September 2022, which can be accessed at: 10.1186/ISRCTN35268189.

### Data analysis

We tested for univariate between-group differences in demographics, socioeconomic and health status, and symptom score between the post-COVID group and control group using t-test, chi-square test or their non-parametric equivalent. To differentiate between post-covid subjects with a high or low symptom load, we used weighted Wasserstein distance to define a cut-off point, such that the group with a symptom number below the cut-off point (the ‘low symptom load group’) had the closest level of mental health and sleep distress, HRQoL and cognitive task performance as compared to the matched control group. The high symptom load group was then defined as the group with a symptom number above the cut-off point. To calculate the Wasserstein distance, the weights for each measurement were taken from the regression coefficients of the logistic regression model that separated the post-COVID subjects from the control group. We then performed a univariate between-group differences analysis in demographics, socioeconomic and health status, and symptom score between the high and low symptom load groups using t-test, chi-square test or their non-parametric equivalent. We used a multivariable regression model to look for potential predictors after adjusting for potential confounders.

To explore the relationships among chronic post-COVID neuropsychiatric symptoms and their clustering patterns, we built a regularised partial correlation network based on the data from the post-COVID group in relation to self-reported neuropsychiatric symptoms [20, 21]. To minimise the instability of the network, we excluded symptoms suffered by fewer than or equal to 20 people (out of a sample of 223 people). After network estimation, we used the *walktrap algorithm* to discover the communities/clusters within the symptom network. To validate the robustness of the detected communities, we used the *community assortativity* (r_com) metric, a bootstrapping procedure, to measure the robustness of community assignments done by the *walktrap algorithm*. Community assignments are deemed to be robust if r_com is larger than 0.5 [22]. We used R packages of *bootnet*, *IsingFit, igraph, ggraph* and *plyr* to perform the network estimation process, and *asnipe* and *assortnet* for assortativity metric estimation. For details about the network estimation and validation procedures, please see Supplementary Method.

## Results

We recruited 223 post-COVID cases and 224 non-COVID control subjects (see recruitment flowchart in Supplementary Material). For the post-COVID group, the mean duration from index infection to assessment was 757 days (range = 371–1105 days). The majority of the post-COVID subjects had no known pre-COVID physical or mental comorbidities (80.7 and 95.5%, respectively) (see Supplementary material for the full list of symptoms and their frequency), and the majority of them (70%) suffered from no or mild respiratory symptoms during the acute phase of their first COVID infection. 25% of the subjects had suffered from at least one re-infection. There were no statistically significant differences in key demographics and pre-pandemic socioeconomic and health status between the post-COVID and control groups (Table 1).

**Table 1:** Comparisons of baseline characteristics, post-pandemic changes of health and socioeconomic status, infection and vaccination status between subjects of the post-COVID group and control group, and between high post-COVID neuropsychiatric symptom load group and low symptom load group, respectively.

Variables	Post-COVID group	Control group	*P* value	High symptom load group	Low symptom load group	*P* value
**Baseline Characteristics**						
Age, year mean (SD)	42.2 (14.4)	41.6 (13.7)	0.65	44.8 (14.0)	41.1 (14.4)	0.083
Female sex, %	60.5%	61.6%	0.82	61.2%	60.3%	0.90
Non-Chinese ethnicity, %	2.7%	2.7%	0.99	0%	3.8%	0.10
Tertiary education level, %	54.7%	62.1%	0.12	49.3%	57.1%	0.28
Pre-pandemic deprivation index, median (IQR)	1.0 (3)	0.5 (2)	0.098	1.0 (4)	1.0 (2)	0.065
Pre-pandemic no. of medical conditions, mean (SD)	0.27 (0.64)	0.28 (0.65)	0.94	0.30 (0.58)	0.26 (0.66)	0.31
Any psychiatric conditions pre-pandemic, %	4.5%	4.9%	0.83	4.5%	4.5%	0.10
Pre-pandemic suicidal ideation	4.5%	6.2%	0.42	3.0%	5.1%	0.30
**Post-pandemic changes in health and socioeconomic status**						
Change in deprivation index, median (IQR)	0 (0)	0 (0)	0.89	0 (0.5)	0 (0)	0.17
No. of new medical conditions, mean (SD)	0.14 (0.42)	0.05 (0.24)	0.0062	0.25 (0.50)	0.10 (0.37)	0.0022
Any new psychiatric conditions, %	2.7%	3.1%	0.79	7.5%	0.6%	0.01
New suicidal ideation, %	7.2%	4.5%	0.22	16.4%	3.2%	0.001
**Infection and vaccination status**						
Any COVID vaccination before infection, %	11.2%	N/A	-	4.5%	14.1%	0.038
>1 infection, %	26.0%	N/A	-	22.4%	27.6%	0.42
First infection severity, %						0.63
Mild	70.0%	N/A	-	65.7%	71.8%	-
Moderate	18.8%	N/A	-	22.4%	17.3%	-
Severe or ICU admission	11.2%	N/A	-	11.9%	10.9%	-

The high symptom load group is defined as subjects with four or more chronic neuropsychiatric symptoms, while the low symptom load group is defined as subjects with fewer than four persistent neuropsychiatric symptoms. All comparisons between groups were conducted with non-parametric statistical tests, including Mann–Whitney *U*-test, Pearson’s Chi-squared test and Fisher’s exact test.

*SD* standard deviation, *IQR* interquartile range.

The differences in the level of depressive, anxiety, post-traumatic stress and insomnia symptoms between the post-COVID and control groups did not reach statistical significance. The post-COVID group, however, had significantly more fatigue symptoms than the control group (bimodal scoring; median = 3.0 vs 2.0, *U* value = 29830.5, *p* < 0.001) despite a similar daily sleep duration. The HRQoL measures of the two groups were comparable. In relation to app-based cognitive tasks, there was no statistically significant difference between the two groups in terms of their performance in the PVT and N-back tasks. The post-COVID group performed worse in the DSST (median of total accurate count = 58.0 vs 60.5, *U* value = 21565.5, *p* = 0.013) and the alternate finger-tapping task as compared to the control group (median of total accurate count = 34.0 vs 41.0, *U* value = 21540.0, *p* = 0.012) (Table 2).

**Table 2:** Comparison of mental health measures, cognitive task performance and health-related quality of life outcome between subjects of the post-COVID group and control group, and between high post-COVID neuropsychiatric symptom load group and low symptom load group, respectively.

Variables	Post-COVID Group		Control Group			High symptom load group		Low symptom load group		
	Median	IQR	Median	IQR	*p* value	Median	IQR	Median	IQR	*p value*
Mental Health Measures										
PHQ-9	4.0	6.0	4.0	5.0	0.50	8.0	6.5	3.0	6.0	<0.001
GAD-7	3.0	7.0	2.0	5.0	0.063	6.0	6.0	2.0	5.0	<0.001
ISI	7.0	9.0	6.0	8.0	0.18	11.0	7.5	5.0	8.0	<0.001
CFS	3.0	6.0	2.0	5.0	<0.001	8.0	5.0	2.0	5.0	<0.001
IES-R	10.0	17.0	8.0	13.0	0.22	19.0	17.5	7.0	13.3	<0.001
Daily sleep duration, hr	6.9	1.5	6.9	1.3	0.21	6.3	1.7	7.1	1.2	<0.001
**Cognitive task performance**										
PVT correct reaction time, seconds	0.3	0.1	0.3	0.1	0.93	0.3	0.1	0.3	0.1	0.26
DSST correct count	58.0	18.0	60.5	16.0	0.013	57.0	16.0	58.5	20.3	0.91
Two back correct count	59.0	16.0	58.0	19.0	0.48	58.0	16.0	60.0	16.3	0.69
Finger-tapping task correct count	34.0	39.5	41.0	33.0	0.012	33.0	41.0	34.0	38.5	0.59
**Health-related quality of life**										
WHOQOL-BREF domains										
Physical health	67.9	25.0	67.9	18.8	0.43	57.1	21.5	71.4	21.4	<0.001
Psychological health	54.2	25.0	54.2	21.9	0.21	45.8	23.0	58.3	20.9	<0.001
Social health	58.3	16.7	58.3	16.7	0.88	50	20.8	58.3	25	0.0078

The high symptom load group is defined as subjects with four or more persistent neuropsychiatric symptoms, while the low symptom load group is defined as subjects with fewer than four persistent neuropsychiatric symptoms. All comparisons between groups were conducted with non-parametric statistical test, including Mann–Whitney *U*-test, Pearson’s Chi-squared test and Fisher’s exact test.

*IQR* interquartile range, *PHQ-9* patient health questionnaire-9, *GAD-7* generalised anxiety disorder assessment, *ISI* insomnia severity index, *CFS* Chalder fatigue scale, *IES-R* revised version of the impact of event scale, *hr* hour, *PVT* psychomotor vigilance task, *DSST* digit symbol substitution test, *WHOQOL-BREF* World Health Organisation quality of life scale.

The post-COVID group had more newly diagnosed medical conditions since the beginning of the COVID pandemic (mean = 0.14 vs 0.05, *U* value = 26782.5, *p* = 0.0062), but not psychiatric diagnoses (see Supplementary material for the full list of symptoms and their frequency). They also had more days of hospitalisation in the past year (median = 0 vs 0, mean = 0.9 vs 0.2, *U* value = 26370.5, *p* = 0.048) but not more medical consultations. The post-COVID and control groups did not have statistically significant changes in socioeconomic status, as reflected by changes in the Deprivation Index and subjects’ employment status.

Among patients who were infected by the SARS-CoV-2 virus, 60% reported at least one neuropsychiatric symptom which appeared during the acute or early post-recovery period of their first COVID infection and has persisted until now. The most frequent complaints were memory problems (50.2%), fatigue (31.8%), inability to concentrate (30.5%), anxiety (21.1%), insomnia (20.6%), post-traumatic stress (PTS) (18.4%, as measured by IES-R), daytime sleepiness (17.5%) and feeling depressed (15.2%) (Table 3).

**Table 3:** List of top ten COVID neuropsychiatric symptoms (*N* = 223).

Symptoms	Frequency	Percentage
Memory problems	112	50.2%
Fatigue	71	31.8%
Inability to concentrate	68	30.5%
Feeling anxious	47	21.1%
Insomnia	46	20.6%
PTSD symptoms	41	18.4%
Daytime sleepiness	39	17.5%
Feeling depressed	34	15.2%
Headache	30	13.5%
Loss of interest or pleasure	29	13.0%

### Defining symptom load groups

**Fig. 1:**
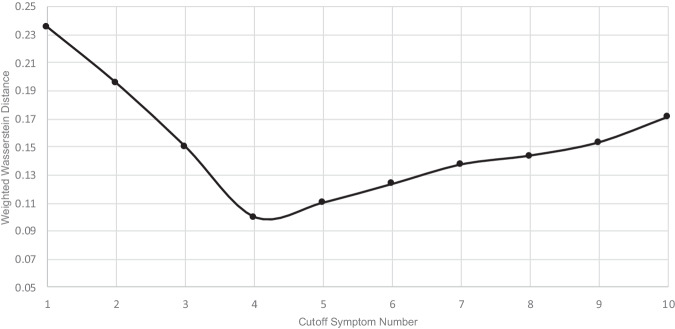
Optimal cut-off number of symptoms for defining the high/low symptom load groups. The weighted Wasserstein distance between the group with their number of symptoms below different cut-offs and the matched control group. The cut-off symptom number that results in the shortest distance is the optimal cut-off to define the high/low symptom load groups.

The levels of depression, anxiety, fatigue, insomnia, PTS, as well as HRQoL measures, but not cognitive performance, worsened as the number of chronic neuropsychiatric symptoms reported increased (see Supplementary material). The weighted Wasserstein distance analysis showed that post-COVID subjects with less than four persistent neuropsychiatric complaints were closest to the matched control group in terms of the level of mental and sleep problems, HRQoL and cognitive task performance (Fig. 1). Thus, post-COVID subjects with less than four persistent symptoms were defined as the low symptom load group, while those with four symptoms or more were defined as the high symptom load group.

According to this cut-off, 67 of our subjects (30%) had a high symptom load (Table 2). There were no statistically significant differences in the baseline characteristics of the high and low symptom load groups, although the mean age of the high symptom load group was nominally higher (mean = 44.8 vs 41.1, *U* value = 5993.2, *p* = 0.083), and the difference in pre-COVID levels of social deprivation between the groups approached statistical significance (median = 1.0 vs 1.0, *U* value = 6008.5, *p* = 0.065). The median number of pre-morbid medical conditions and the presence of known psychiatric conditions in the two groups were similar, though the high symptom load group had a higher number of persistent non-neuropsychiatric symptoms after the acute phase of COVID infection (median = 6 vs 0, *U* value = 9577.5, *p* < 0.001) (Table 1).

The high symptom load group had a statistically significantly higher number of newly diagnosed medical conditions (median = 0 vs 0, mean = 0.3 vs 0.1, *U* value = 5990.0, *p* = 0.002). The most common new medical conditions among the high symptom load group are chronic pain (10.4%), hypertension (7.5%), and heart disease (4.5%). The high symptoms load group also has a statistically significantly higher incidence of new psychiatric conditions (7.5 vs 0.6%, Fisher’s exact test, *p* = 0.01) and suicidal ideas (16.4 vs 3.2%, Fisher’s exact test, *p* = 0.001). Additionally, they had more medical consultations (median = 3.0 vs 1.0, *U* value = 6787.5, *p* < 0.001) and days of hospitalisation (median = 0 vs 0, mean = 0.7 vs 1.0, *U* value = 5915.5, *p* = 0.007) in the past year.

The high symptom load group was less likely to have received any COVID vaccine prior to their first SARS-CoV-2 infection (4.5 vs 14.1%, Fisher’s exact test, *p* = 0.038) when compared to the low symptom load group, but the two groups were similar in terms of the proportion of moderate to severe/critical severity of the subjects’ COVID infection during the acute phase (34.3 vs 28.2%, Pearson’s chi-square = 0.84, *p* = 0.43) and whether they had single or multiple COVID infections (22.4 vs 27.6%, Pearson’s chi-square = 0.65, *p* = 0.51).

A multivariable logistic regression performed using age, gender, ethnicity, tertiary education, pre-pandemic Deprivation Index (log-transformed), number of pre-pandemic medical comorbidities, presence of pre-pandemic psychiatric diagnosis, receipt of COVID vaccination prior to first infection, severity of index COVID infection and single or multiple infections as predictors showed that only the pre-pandemic Deprivation Index (adjusted odd ratio = 1.55, 95% CI = 1.04–2.31, *p* = 0.032) and receipt of any COVID vaccination prior to first infection (adjusted odd ratio = 0.26, 95% CI = 0.07–0.92, *p* = 0.037) were statistically significant predictors for the high symptom load group. These two factors also predicted the count of chronic post-COVID neuropsychiatric symptoms of the subjects in a generalised negative binomial regression model using the same variables (see Supplementary material).

### Network analysis and symptom clustering

**Fig. 2:**
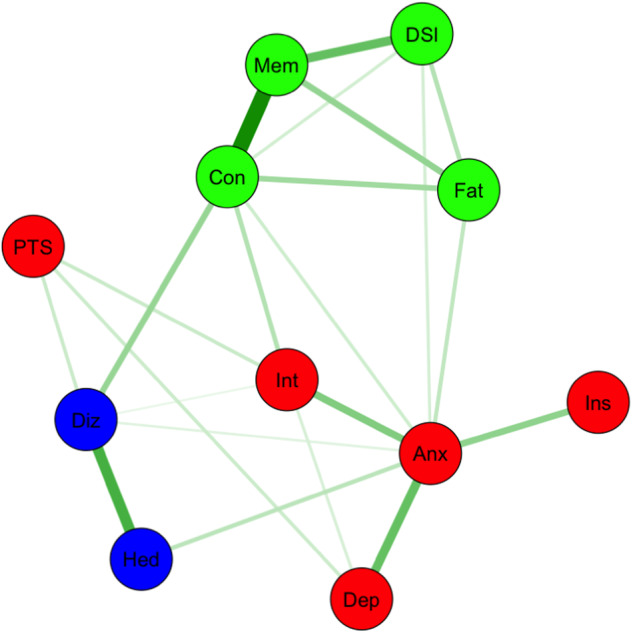
Partial correlation network of post-COVID chronic neuropsychiatric symptoms. Fat: fatigue, DSl daytime sleepiness, Mem memory problems, Con Inability to concentrate, Anx feeling anxious, Ins insomnia, Dep feeling depressed, Int loss of interest or pleasure, PTS COVID-related post-traumatic stress symptoms, Diz dizziness, Hed headache. The colour of the node represents the cluster they belong to.

Our network model identified three distinct clusters/communities: (1) an anxiety-depression cluster containing symptoms of anxiety, insomnia, loss of interest or pleasure, depression and PTSD; (2) a cognitive complaint-fatigue cluster containing fatigue, inability to concentrate, memory problems and daytime sleepiness; and (3) a dizziness-headache cluster (Fig. 2). The r_com of the network was 0.68, indicating our network model likely contained discrete clusters and that the *walktrap algorithm* had reliably detected them.

Multivariable negative binomial regression models using the same predictors as our logistic regression above suggested that the pre-pandemic Deprivation index (log-transformed) positively predicts the number of chronic symptoms in both the anxiety-depression (b = 0.40, *p* = 0.0002) and cognitive complaint-fatigue clusters (b = 0.23, *p* = 0.025), but receipt of any COVID vaccination before the index infection negatively predicts only the number of symptoms from the cognitive complaint-fatigue cluster (b = −0.79, *p* = 0.0018) and not that of the anxiety-depression cluster (b = −0.39, *p* = 0.28).

## Discussion

To the best of our knowledge, our study is the first that examines chronic neuropsychiatric symptoms persisting beyond one year after the first SARS-CoV-2 infection using (i) non-COVID control data to define a threshold for high/low symptom load and (ii) network analysis to explore the structure of such symptoms, as well as the clinical and socioeconomic risk factors of symptom clusters.

### Higher levels of fatigue and worse performance in neurocognitive tasks among post-COVID subjects

The post-COVID group had a higher level of fatigue than the matched control group after more than one year from the index infection, a finding which was not explained by depression, anxiety, PTS, or insomnia. This result echoes existing evidence that fatigue is one of the most reported PACS symptoms in the post-infection era [23], with the additional implication that fatigue may be more persistent than mood, anxiety, sleep or PTS symptoms among post-COVID patients. The post-COVID group also performed worse than the matched control group in the DSST and alternate finger-tapping tests. The DSST is a nonspecific psychomotor speed test involving multiple domains, and is sensitive in detecting cognitive dysfunction [24]. The alternate finger-tapping task is also a psychomotor speed test, though one that requires less cognitive processing. In the absence of any impairment in the subjects’ attention and working memory task performance, we are unable to pinpoint the specific deficient neurocognitive domain(s) behind the psychomotor speed impairment among the post-COVID group.

### Increase in medical diagnoses and medical resource utilisation among post-COVID subjects

Post-COVID subjects had an increase in medical diagnoses since the COVID pandemic began, and showed an increase in hospitalisation in the past 1 year. This is consistent with other reports suggesting that there has been an increase in the incidence of some medical conditions after SARS-CoV-2 infection [9]. The post-COVID group did not show an increase in post-COVID psychiatric diagnoses and new suicidal ideas, however. This suggests that, as a group, there is no evidence of clinically concerning mental health deterioration after infection, despite an increase in new diagnoses of physical conditions. The socioeconomic status of post-COVID subjects as compared to the matched control group did not worsen since the COVID pandemic began, and the HRQoL of both groups were comparable. Overall, these results reflect that, as a group, there has been some change in the health status of post-COVID subjects, even though this did not have a significant impact on their social and vocational functioning and HRQoL.

### Post-COVID subjects with multiple neuropsychiatric complaints had poorer mental health and HRQoL

If we look at the comparison between those with a high load of persistent neuropsychiatric symptoms and those with a low load, however, the two groups have pervasively different post-COVID health trajectories despite similar pre-COVID health. This is clearly reflected by the new incidence of medical and psychiatric diagnoses, the emergence of suicidal ideation, increased medical consultations and hospitalisations and poor current mental health and worse current HRQoL of the post-COVID group, in the absence of significant pre-COVID health and socioeconomic discrepancies as compared to the matched control group. While we cannot tell the direction of any potential causal relationship between the neuropsychiatric and other aspects of health based on our data, given that this relationship may be bidirectional, or the two may share similar pathophysiology, the bottom line is that the presence of multiple persistent post-COVID neuropsychiatric complaints reflected quantifiable and clinically relevant mental health distress. Notably, however, cognitive performance did not differ between the high and low symptom load groups. This can reflect the relationship between cognitive impairment and chronic post-COVID neuropsychiatric symptoms loading is non-linear, or the subjective cognitive complaints do not reflect objective impairment. Yet, it could also have been a result of the limitations of the tools used. For example, because the cognitive tasks were completed via a mobile phone app in an uncontrolled environment, this may have increased the variance in performance. The range of cognitive functions that our test panels could cover was also limited.

### Lack of vaccination and pre-pandemic material deprivation predicts high number of chronic neuropsychiatric complaints but not index infection severity

Two of our three hypothesised risk factors, namely vaccination status before first COVID infection and pre-pandemic level of material deprivation as measured by the Deprivation Index, were found to be predictive factors of a high load of persistent neuropsychiatric symptoms. While previous studies have reported that COVID vaccinations reduce PACS [10], our study is the first to date that demonstrates that any COVID vaccination before infection reduces the risk of chronic neuropsychiatric symptoms even more than one year after infection, with this protective effect not being one that is mediated through reducing symptom severity or protection against re-infection. Thus, it is possible that vaccination reduces the dysregulated response of the immune system during SARS-CoV-2 infection, which is hypothesised to be the underlying pathophysiology of PACS. In this way, our findings highlight a further public health benefit of COVID vaccinations beyond reducing short-term morbidity and mortality.

Our results also suggest that socioeconomic factors cannot be overlooked when we are considering persistent post-COVID neuropsychiatric complaints. Material deprivation has been demonstrated to be a common contributing factor to mental health distress at the population level (Chung et al., 2021). In the context of the COVID pandemic, the association of poor COVID outcomes with health inequalities has been a recurrent theme [25, 26]. It is, therefore, not surprising that material deprivation was found to predict chronic post-COVID neuropsychiatric symptoms. While other studies have shown similar results, the limitations in the designs of such studies, namely in terms of the potential for reverse causality, have made it difficult to draw such conclusions straightforwardly from their results. A study from Hong Kong, for example, reported financial worry as one of the predictors of post-COVID mental health trajectory [27]. Because they only measured the subjects’ evaluation of their financial situation post-COVID, however, there is difficulty determining the direction of causality given that a self-reported concern about financial status can be secondary to and a result of poor mental and physical health. A US-based study found that life stressors post-COVID are associated with neuropsychiatric outcomes 12 months post-infection [28], but the causal direction of this association is again unclear. To minimise concerns regarding reverse causality in our study, we collected pre-pandemic Deprivation Index data.

Contrary to our hypothesis, the severity of the index infection did not predict whether there would be a high or low load of chronic neuropsychiatric symptoms post-COVID. Available evidence on the ability of infection severity to predict PACS has not been consistent [27, 29–32]. There is convergent evidence, however, that persistent PACS can occur in individuals with mild infections.

### Embracing the heterogeneity among persistent neuropsychiatric complaints

Our network analysis suggested that the common chronic post-COVID neuropsychiatric symptoms can be grouped into two major (anxiety-depression and cognitive complaint-fatigue) and one minor (headache-dizziness) clusters, a finding which supports our hypothesis that such symptoms form clusters. These clusters suggest that there could be different sub-syndromes or even disorders under the umbrella term of persistent neuropsychiatric complaints.

We also found that socioeconomic disadvantage increased the symptom load of both major clusters, but that vaccination only significantly decreased the symptom load of the cognitive complaint-fatigue cluster. This difference in how predictive the factors were gives additional support to the idea that neuropsychiatric symptoms can be further categorised into subgroups, as different sub-syndromes may have different underlying risk factors and mechanisms.

Previous studies have also used clustering algorithms to categorise individuals with PACS into subgroups [33, 31]. These algorithms usually use a mix of self-reported symptoms, assessment results, subject demographics and clinical factors to perform the clustering, whereas our approach is to first identify the clustering of symptoms before we examine the unique, associated factors of the discovered clusters. We argue that our approach is clearer phenomenologically because it reveals the relationship among symptoms independent of confounding sociodemographic factors. The key problem with any clustering result, however, is its generalisability. Although we have conducted extra bootstrapping procedures to demonstrate the stability of our clustering results, the further replication of these results using an independent cohort is important to support the generalisability of the clustering. To the best of our knowledge, Peter et al.’s study (Peter et al., 2022) is relatively comparable to ours in terms of study approach. Despite substantial differences in sampling, measurement and statistical tools employed, the results of their clustering analysis were largely consistent with ours: they found a cluster consisting of headache and dizziness, and another consisting of sleep, anxiety and depression.

### Strengths and limitations

The key strength of this study is that it was conducted in accordance with a pre-registered, open-access protocol with pre-determined hypotheses. Another strength is that our assessment and analysis covered comprehensive demographic, clinical and socioeconomic factors, as well as symptoms and cognitive measures. The first and the key limitation of this study is the use of the convenient sampling method, which limits the certainty of any epidemiological inference. The second major limitation is the cross-sectional design of the study. While we used the pre-COVID socioeconomic and health status of the subjects for case-control matching and regression analysis, so as to avoid bias introduced by changes in their physical and mental health due to COVID, the pre-COVID socioeconomic and health status data were based on recall, and thus may be affected by recall bias. The modest sample size also limits statistical power. The third limitation is that the infection status of the subjects was based on self-report and was not verified by our own testing, which could have affected the reliability of the data. However, since Hong Kong implemented a ‘zero-COVID’ policy with very stringent surveillance prior to the Omicron wave in early 2022, the subjects’ understanding of their infection status is likely accurate. The fourth limitation is that we relied on self-reported checklists and symptom scales only, and did not conduct clinical interviews or use clinician-rated instruments. Lastly, we did not correct for multiple comparisons in our statistical tests, which increases the risk of false positive results. While using pre-registered outcome measures can partially mitigate the problem of false positivity, the evidence should only be regarded as preliminary.

## Conclusions

Our study suggests that the lack of COVID vaccination and a higher level of material deprivation before the COVID pandemic predicts a higher load of chronic post-COVID neuropsychiatric symptoms that persists for more than one year post-infection. This highlights the complex interplay of biological and socioeconomic factors that contribute to chronic post-COVID neuropsychiatric symptoms. Our analysis also suggests heterogeneity among chronic post-COVID neuropsychiatric symptoms, with two major symptom clusters (anxiety-depression and cognitive complaint-fatigue) and one minor cluster (headache-dizziness). Further study into chronic neuropsychiatric sequelae should take into account the heterogeneity and complexity of its phenomenology and aetiology.

### Supplementary information

Supplementary Materials

## Data Availability

The data that support the findings of this study will be openly available after the paper is published.
